# Comparative analysis of text-based plagiarism detection techniques

**DOI:** 10.1371/journal.pone.0319551

**Published:** 2025-04-08

**Authors:** Muhammad Sajid, Muhammad Sanaullah, Muhammad Fuzail, Tauqeer Safdar Malik, Shuhaida Mohamed Shuhidan

**Affiliations:** 1 Department of Computer Science, Air University, Islamabad, Pakistan; 2 Computer Science Department, NFC Institute of Engineering and Technology, Multan, Punjab, Pakistan; 3 Department of Information & Communication Technology, Bahauddin Zakariya University, Multan, Punjab, Pakistan; 4 Centre for Research in Data Science, Computer and Information Sciences Department, Universiti Teknologi Petronas, Perak, Malaysia; University of the Witwatersrand Johannesburg Faculty of Health Sciences, SOUTH AFRICA

## Abstract

In text analysis, identifying plagiarism is a crucial area of study that looks for copied information in a document and determines whether or not the same author writes portions of the text. With the emergence of publicly available tools for content generation based on large language models, the problem of inherent plagiarism has grown in importance across various industries. Students are increasingly committing plagiarism as a result of the availability and use of computers in the classroom and the generally extensive accessibility of electronic information found on the internet. As a result, there is a rising need for reliable and precise detection techniques to deal with this changing environment. This paper compares several plagiarism detection techniques and looks into how well different detection systems can distinguish between content created by humans and content created by Artificial Intelligence (AI). This article systematically evaluates 189 research papers published between 2019 and 2024 to provide an overview of the research on computational approaches for plagiarism detection (PD). We suggest a new technically focused structure for efforts to prevent and identify plagiarism, types of plagiarism, and computational techniques for detecting plagiarism to organize the way the research contributions are presented. We demonstrated that the field of plagiarism detection is rife with ongoing research. Significant progress has been made in the field throughout the time we reviewed in terms of automatically identifying plagiarism that is highly obscured and hence difficult to recognize. The exploration of nontextual contents, the use of machine learning, and improved semantic text analysis techniques are the key sources of these advancements. Based on our analysis, we concluded that the combination of several analytical methodologies for textual and nontextual content features is the most promising subject for future research contributions to further improve the detection of plagiarism.

## Introduction

The ease with which knowledge may be shared through global interactive communication platforms has prompted writers to conduct targeted online searches for information [1]. This talent has had a negative effect in that individuals have attempted to take credit for their work by copying ideas or research without giving due credit [2]. It has been noted, especially in the scholarly community. One of the most significant issues is plagiarism detection, which has several focuses, including text mining, academic literature standards, and natural language processing (NLP) [3]. There are still a lot of unsolved problems about borderline sets and standards. To provide the numerous advantages of plagiarism detection, some are rather simple, while others need the application of complex algorithms and scientific ideas [4]. The prevalence of plagiarism in academic settings has increased due to its discovery in several student works, such as papers, assignments, projects, and more. Academic plagiarism is defined as using ideas, terminology, or structures without properly citing the source [5]. While there are differences in the ways that students approach plagiarism, the most extreme cases involve the complete rewriting of original information [6]. Additional strategies include rewording material via online paraphrasing services, replacing terms with synonyms, and partially rephrasing text through changes to grammatical structures [7]. Academic plagiarism is one of the most serious forms of misbehavior since it compromises the acquisition and evaluation of competencies, in violation of ethical standards [8].

Plagiarism Detection (PD) is one of the most important problems in text analysis. Its goal is to locate instances of illegal duplication or content infringement within a single document [9]. The design of plagiarism detection is predicated on the notion of analyzing a single document independently, without the need to compare it to additional sources or documents [10,11]. Although plagiarism by researchers in papers and by students in projects is not a new problem, it has become more problematic due to the ease with which information may be “copied and pasted” from journals and other online sources [12]. Although using other people’s content can be intentional, it is usually an unintentional error. publishers, instructors, examiners, and others can identify plagiarism more quickly and readily with the software programs already on the market [13]. Additionally, they can give up relying on their ability to spot parallels in previously released content [14]. Plagiarism detection software can assess student and authored papers in a matter of minutes by comparing them to previously published works.

To ensure text authenticity across a range of applications, Open AI classifier tools have recently come to be depended upon for differentiating between content generated by AI and writing done by humans. For example, Open AI, the company that created ChatGPT, unveiled an AI text classifier that helps users distinguish between essays written by humans and those written by AI [15]. Based on the possibility that a document is artificial intelligence (AI) generated, this classifier divides texts into five categories: very unlikely, unlikely, ambiguous, probably, and likely AI-generated [16]. Although not all forms of human written text are included in the training data, the OpenAI classifier has been trained on a wide variety of texts. Furthermore, the tests conducted by the developers reveal that the classifier incorrectly identifies 26% of the AI-written text as “likely AI-generated” while accurately labelling 9% of the human-written content as AI-generated [17]. Therefore, rather than depending solely on the classifier’s results to determine AI-generated material, Open AI recommends users consider the results as supplemental information. Writer.com’s AI content detector is one of the several AI text classifier tools available. It highlights the usefulness of AI-generated content for content marketing and provides a restricted application programming interface API-based solution for recognizing it. With a 99% accuracy rate, Copyleaks is an AI content detection system that integrates with numerous learning management systems and APIs. The authors Emi, Bradley, and Spero, Max developed GPTZero [18], an OpenAI classifier tool designed to identify AI-generated content in student submissions and prevent AI plagiarism in educational settings.

Selecting the most trustworthy and efficient plagiarism detection system these days can be challenging due to the abundance of options [19]. Consequently, this paper presents the results of a survey-based study for selecting an efficient academic plagiarism detection method. Plagiarism checkers are successful in identifying extrinsic plagiarism each year, while plagiarism only depends on stylometric functionality that is examined by using the arrangement of the papers, according to a quantitative research study on a variety of plagiarism types across a broad range of plagiarised scripts [20]. A thorough analysis of the classification of plagiarism-checking methods was conducted, concentrating on textual characteristics, organizational characteristics, semantic structures, candidate-information extraction prototypes, and plagiarism-finding procedures. Concept plagiarism appears in the downstream hierarchical structure of clever plagiarism kinds [21] because it lacks the textual semantics required to transmit the concept and the format’s localization of perspective. The primary objective of this survey is to comprehensively review and evaluate the state of the art in terms of plagiarism detection techniques [22,23]. This survey also aims to clarify the difficulties that are unique to low-resource languages and suggest possible lines of inquiry for further research in plagiarism detection, which will ultimately improve plagiarism detection techniques and lessen the likelihood of illegal content replication [24].

## Literature review

Literature reviews have become a vital tool for thoroughly investigating and understanding a range of topics. Numerous perceptive review studies have been carried out in a variety of domains [25], shedding light on important issues [26]. These reviews frequently fit into different categories, such as meta-reviews or mapping studies [27], narrative or conventional reviews [28], and systematic literature reviews [29]. A review of the literature is provided in this study, which explores the topic of plagiarism detection and offers a fresh viewpoint and analysis on the subject. A noteworthy advancement in this subject occurred in the middle of 2019 with the release of the GROVER model [30], which can both create and identify fake news. GROVER, which surpassed previous deep pre-trained models, boasted a 92% accuracy rate in identification and had access to 5000 of its own created articles in addition to infinite genuine news items. The Giant Language Model Test Room, or GLTR tool [31] was made available in June 2019. The open-source tool GLTR finds generation artifacts from different sampling methods used in language models by looking at texts made by models like Generative Pre-trained Transformer-2 (GPT-2) using some different baseline statistical methods. Subsequently that year, OpenAI released a customized GPT-2 detector [32], which improved the Roberta model [33].

In a study, [12] the authors Altheneyan and Menai, conducted a critical analysis of the methods currently used for paraphrase detection and automated plagiarism detection results. It explained the categories of strange events, the fundamental approaches, and the sets of characteristics that each method made use of. It evaluated and investigated how well plagiarism-detection methods that recognize paraphrases in benchmark corpora perform. The main discovery was that word overlapping and structural interpretations are feature subcategories that help support vector machine (SVM) in paraphrase identification and plagiarism detection in corpora produces the best presentation outputs. Deep learning techniques are the most interesting area of research in this discipline, according to a study [34] on their effectiveness. A unique model for creating and identifying fraudulent internet reviews was created in 2020 [35]. This novel strategy used a fine-tuned BERT model as a classifier for the detection phase, combining the review generation capabilities of GPT-2. In a study [36], the authors Uchendu et al. investigated the difficulty of differentiating human-written texts from those produced by neural network-based language models in the same year. Their focus was on three different authorship attribution problems: determining whether two texts were generated by the same algorithm, determining whether a piece was written by a machine or by a human, and identifying the exact neural algorithm that generated a given text. To conduct their empirical studies, they used writings produced by eight different models Conditional Transformer Language Model (CTRL) [37], Cross-lingual Language Model (XLM) [38], Generalized Autoregressive Pretraining for Language Understanding (XLNET) [39], and Plug and Play Language Model (PPLM) [40] as well as texts authored by people. The study found that while the majority of text generators continue to provide content that can be distinguished from Human-Written Text (HWT), other models such as GPT2, GROVER, and FAIR deliver higher-quality outputs that cause machine classifiers to become confused more often.

As part of the “Tweep Fake” project [41,42], a detector was created by the authors Fagni et al. and Jawahar et al. in 2021 to detect deepfake tweets. The first authentic dataset of deepfake tweets on Twitter was created as part of this project. The research team collected and analyzed tweets from 23 bots that imitated 17 real user accounts using a range of content-generating techniques, including GPT-2, RNN, LSTM, and others. In 2022, a different study [43] revealed a neural network-based detector that combines textual data with explicit factual information. This is made possible via entity-relation graphs, which are captured in the text as interactions between different entities and relationships and are encoded by a graph convolutional neural network. By reasoning about the facts provided, the goal of the model is to distinguish modified news items from detectors that only use stylometric signals. In a study [44], the authors Guo et al. compare the capabilities of ChatGPT with human specialists and was one of the first studies released in 2023 that comprised datasets generated by ChatGPT and HWT and detectors trained on the same dataset in both Chinese and English. The Human ChatGPT Comparison Corpus (HC3) is the name given to the vast dataset that the researchers gathered. With the help of databases like ELI5 [45] and ChatGPT, which generates replies for these questions, it has about 40,000 questions and answers covering a wide range of topics like psychology, economics, law, and medicine.

The authors of [46] presented a revolutionary watermarking framework for Large Language Models (LLMs) in 2023. Their method embeds a watermark using the output log-likelihood of LLMs at each generation stage, mainly by using a green token list. This technique identifies and governs the outputs of these potent language models ethically by incorporating detectable signals into the generated text that are unseen to human readers. By the paradigm outlined in [46], Guo et al. proposed three significant enhancements to existing watermarking approaches through more investigation into the topic of watermarking [47]. Another important work on the topic of watermarking carried out by the authors Christ et al. in [48], takes a unique task by utilizing cryptography concepts. This novel approach, which is distinct in that it depends on cryptography, guarantees that watermarks incorporated into LLMs stay undetected unless a particular secret key is used. Details of existing plagiarism detection models are given in Table 1. Moreover, the present survey is unique in that it investigates difficulties in great detail, covering general and low-resource language-specific challenges. Among various studies, [49] has noted challenges; however, none have particularly examined the intricacies of tackling PD issues in languages with limited linguistic resources. In response to the heightened demand for upholding rigorous scholarly ethics in higher education, there is a pressing need to ensure the efficacy of plagiarism detection techniques. This study [50] article aims to examine how higher education institutions might enhance their plagiarism detection capabilities through the utilization of Artificial Intelligence (AI).

**Table 1:** Comparison with SOTA plagiarism detection techniques.

Ref	Dataset	Feature Extraction	Detection Techniques	Year
[54]	documents	Syntax-based	MT-DNN	2019
[55]	Urdu blogs	Lexicon-based	Urdu sentiment analyzer	2020
[36]	Human Vs Grover	Lexical and Syntax	NN Models	2020
[56]	S24,S1000	Stylometric-based	Classification algorithms	2021
[24]	English-Arabic corpora	Semantic-based	NN Classifiers	2022
[15]	Wikipedia Articles	Perplexity and Semantic	XGBoost-RF	2023
[2]	Student & ChatGPT Essays	Statistical-based	AI Content Detector	2023
[57]	Comparison Corpus (HC3)	Cosine similarity	Contrastive Loss Function	2023
[19]	PAN 2013-14	Lexical &Syntax	SVM-Chi-square	2023
[58]	XSum	Log probabilities	DetectGPT	2023
[18]	Open Source	Hard negative mining	Transformer-based NN	2024
[59]	HWT and AIGT	Semantic & Syntax	XLM-R & ELECTRA	2024
[21]	SNLI-MSRP	Automated feature extraction	Artificial Bee Colony	2024

The authors in a study [51], claimed that a 2023 survey examined 3,017 high school and college students. It was discovered that nearly one-third admitted to utilizing ChatGPT for homework aid. The emergence of Large Language Models (LLMs) like ChatGPT and Gemini has increased academic dishonesty. Students can now fulfill their tasks and examinations merely by requesting solutions from a language model, circumventing the necessary effort for learning. Moreover, it is concerning because instructors lack the appropriate instruments for detection. Primarily, plagiarism detection systems are employed in the education sector; however, they are also useful in other fields such as journalism and media, business, and the creative arts. The Turnitin system is designed to reduce textual similarities in articles and scientific papers. Turnitin, a prevalent plagiarism detection software, offers a mechanism to identify and mitigate plagiarism. Nonetheless, it is crucial to tailor it to the environment and particular requirements of Islamic Religious Education programs at private Islamic universities. The proposed method [52] entails the integration of Turnitin with Islamic literary databases and the modification of detection algorithms to identify religious texts. Plagiarism has become a prominent topic of discussion in higher education institutions recently. Turnitin text-matching software has been extensively used by numerous academic institutions in Ghana as a means to enhance the academic writing of students and professors, as well as to identify instances of plagiarism. Despite extensive research on attitudes, motivations, and demographic factors associated with academic dishonesty, there has been limited empirical investigation into students’ actual knowledge of plagiarism and their experiences with text-matching technology, as indicated by a study [53]. Moreover, this survey is unique since it includes a future directions component. This section provides insightful information for future research and development by imagining possible paths and developments for plagiarism detection.

## Survey methodology

To stay true to the main goal of this study, which is to analyze the literature on plagiarism detection, we have compiled knowledge and recommendations from previous approaches and reported in numerous research [25–30]. By utilizing this expertise, we have created acceptable research questions, search methodologies, and clear study objectives. With this method, we efficiently look for and locate pertinent papers in the area of plagiarism detection. The study’s literature review component focused on currently accessible peer-reviewed journal papers on AI detection techniques that differentiate between writings created by AI and texts authored by humans in a variety of disciplines of study. The lack of published, peer-reviewed journal publications in its core area limited its quality assessment, and its searches were thorough but limited by a time frame.

### Ethics statement

Ethical approval was not sought for the present study due to all the data is available.

### Research objectives

The goal of this study was to review articles that were published between 2019 and 2024. It concentrated on how well Artificial Intelligence detection technologies work in identifying text produced by humans and AI. The study’s main focus was on the AI detection technologies used at this time in higher education. Since ChatGPT’s debut and the subsequent spread of AI-powered chatbots, determining which AI detection technologies work better and whether their detection accuracy is dependable have been some of the major issues facing the higher education industry. The research goals of this study are defined as follows:

1. To list and evaluate the most widely used feature extraction methods for plagiarism detection.
2. Examining and contrasting the most popular techniques for detecting plagiarism.
3. To demonstrate how plagiarism detection methods have changed over time.
4. To recognize and investigate the difficulties in identifying plagiarism.

### Research questions

We have prepared a series of relevant research questions, each aimed at delving into distinct facets of plagiarism detection, to tackle the research objectives properly. The following research questions were considered in the study to achieve the goals of the survey:

1. What are the key feature extraction techniques most commonly used?
2. Which methods are most commonly used to detect plagiarism?
3. What does each article aim to achieve?
4. How have methods for detecting plagiarism changed over time?
5. What challenges are there, and how may they be overcome?
6. What are the survey’s primary conclusions and findings?

### Research strategy

We used Google Scholar and Web of Science to conduct a keyword-based automated search [60] to gather the research publications that were part of our survey. The search was restricted to the years 2019 through 2024. We included these foundational publications, regardless of when they were published, to make sure our survey includes all pertinent source material. Online databases, academic social networking sites, and search engines were all used in the search. Different online databases (Google Scholar, PLOS, Taylor & Francis Online, ACM, ScienceDirect, Scopus, and IEEE Xplore Digital Library), two Internet search engines (Google and Microsoft Bing), and ResearchGate comprised these online search platforms. These internet resources were all readily available. Keywords, phrases, and brief sentences of the study’s target area AI detection tools for distinguishing between texts produced by AI and those written by humans were included in search strings. Depending on the search platform, the search strings included truncation symbols like *, or - and Boolean operators like AND or OR. Additionally, variations of these search strings were applied repeatedly. The following search query is used to locate pertinent publications for this investigation.

- “plagiarism” AND “detection”
- “plagiarism” AND “detection” AND “Trends”
- “analysis” AND “stylometric features”
- “Semantic” AND “stylometric features”
- “feature extraction approaches”
- “feature extraction methods”
- “style analysis”
- “grammar analysis”
- “syntax based detection”
- “challenges in plagiarism detection”

### The process of selection

The research selection process is an important step in the literature survey process [61]. For this survey, 189 preliminary studies were gathered from various sources to detect plagiarism. The authors used preset inclusion and exclusion criteria to shortlist the papers during the selection process. Another author was consulted to settle any disagreements, and the inclusion/exclusion standards were improved. The traditional inclusion/exclusion format served as the foundation for the quality assessment criteria that were utilized to determine the eligibility and relevance of the journal articles for this investigation. Journal articles published between 2019 and 2024 met the time-period inclusion criteria. During the designated coverage period, a search and screening procedure was carried out on the fourteen internet search platforms described above to identify journal articles that qualified for inclusion. The web search engines returned 189 articles as a result of this procedure. Of these articles, forty did not satisfy the specified coverage time limit and were thus erased, and 34 were duplicates that were removed as well. Titles and abstracts were examined to narrow down the remaining papers.

1. Search by title: Papers that are unrelated based just on their title are carefully culled in the first step. Numerous papers that were no relevant were present. There were just 115 papers left after this step.
2. Search by Methodology Keywords: In this phase, papers that don’t relate to each other based only on their methodology section keywords rigorously culled. Eventually, only a few pertinent papers remained.
3. Abstract-based search: In this step, the papers are organized for analysis and research approaches after the abstracts of the selected articles were evaluated in the previous step. Just ninety-nine papers were remaining after this.
4. Complete text-based Evaluation: At this point, the articles selected in the previous step are evaluated for their empirical quality. The study’s text has been thoroughly analyzed. A total of forty-five papers were chosen from ninety-nine articles. Four more relevant and qualifying articles were found by the snowballing search that followed. Thus, 49 publications in all were appropriate and qualified for the current investigation.
5. Low-Quality Papers: Excluding papers that were not included in the Google Scholar database was the last step in the research selection process. Additionally, publications that were published without a DOI (Digital Object Identifier) are not included in the analysis.

### Quality assessment criteria

The standard of the selected primary research was evaluated using the following standards. This quality evaluation was carried out by two writers. The study’s main empirical findings are Y-1 and N-0. The study is published in a reputable journal that is selected using the Scientific Journal Ranking (SJR) and the CORE ranking of conferences. For review studies, assessing and guaranteeing methodological quality is crucial. This is true even in cases where the number of review studies in a particular field of study is limited. The authors independently assessed each of the reviewed articles. For every article, sixteen criteria were rated as yes-1 or no-0. The agreement scores between the two raters were calculated using the parameter kappa coefficient K, as referenced in [62]. The disagreements on ratings were settled through discussion and consensus-building [63]. The scoring system [64] developed by the authors Landis et al. and its associated interpretation was used to compute the inter-rater agreement. Inter-rater agreement is a measure of how autonomous raters are when they seek to arrive at the same conclusion when scoring items. This mutual agreement score was considered acceptable because it is between 0.84 and 1.00, which is the almost perfect score range [65,66].

### Results of study selection

To provide answers to the aforementioned research questions, a total of 49 papers were found and examined. Thirteen papers had a score of less than 15%, while 31 papers achieved a score of above 85%. Since they offer some helpful information and were published in reputable journals, some papers have also been included in this analysis. These studies also address significant factors of technology and demography that are directly relevant to plagiarism.

## Scrutiny of survey articles

The primary vulnerabilities associated with systematic literature reviews are inadequacies in the data collection process and the content’s choice, organization, and display. To reduce the possibility of missing any important information, we mainly employed Google Scholar and Web of Science, two of the largest databases for academic literature. We queried the two databases using a multi-stage approach, where the results of each step informed the subsequent one, using keywords that we gradually improved upon to achieve the highest level of coverage. By combining all relevant references of the papers that our keyword-based search had produced, we were able to gather more documents by drawing on the knowledge of domain experts, research paper writers, and literature reviewers on the topic. We also incorporated content-based suggestions from major publishers’ digital libraries, including Elsevier and ACM.

### Datasets

This survey thoroughly examines the standard datasets as given in Table 2 used to evaluate and analyze the field of plagiarism detection. In plagiarism detection, these datasets are essential since they make it easier to compare and assess different detection methods. These databases provide various textual material from many genres and sectors. Researchers can benefit from the PAN datasets https://pan.webis.de/data.html [67], which cover several years and include plagiarism detection scenarios. The Corpus of English Novels (CEN) https://github.com/computationalstylistics/100_english_novels [70] is another noteworthy dataset that provides a special collection for assessing plagiarism detection methods. These datasets work with other sources, such as Wikipedia and a variety of online papers, to improve the accuracy and resilience of plagiarism detection techniques. Their availability enables researchers to create, evaluate, and improve algorithms that can address plagiarism in real-world situations involving a variety of textual sources and styles. Finding copied passages in suspicious work based on an inconsistent writing style is the purpose of the plagiarism detection method.

**Table 2:** Most common used datasets in plagiarism detection.

Ref	Dataset	Language	Referenced in	Size
[67]	PAN 2020	English	[61]	17380
[67]	PAN 2021	English	[60]	13600
[68]	MED	English	[69]	133
[70]	Corpus of English Novels	English	[71]	292
[72]	STS2016	English	[73]	15618
[74]	Urdu Blogs	Urdu	[55]	NA
[75]	InAra	Arabic	[76]	1024

The intrinsic technique, in contrast to the external approach, does not need to compare the dubious material to any possible sources of plagiarism. Of the 1024 texts in the InAra corpus [75], 80% are portions that have been plagiarised to appear authentic. There is an XML file linked to every questionable document that details the length and location of every passage that contains plagiarism. This artificial healthcare dataset was made to give data science, machine learning, and data analysis enthusiasts a helpful resource [68]. The dataset such as given in [77] is notable for its wide range of texts, which include a variety of genres that are common in Urdu writing. It features yearly occasions, prominent citizens of the country, and textual moral teachings to guarantee a representative assortment of Urdu language content. This composition enables a thorough investigation of the complexities involved in plagiarism detection for various document forms. With the use of numerous stylometric parameters, the dataset painstakingly portrays the complex terrain of Urdu writing styles.

### Pre-processing

Careful preprocessing procedures are needed for intrinsic plagiarism detection techniques to eliminate noise and preserve crucial data for analysis [78]. To prevent losing possibly helpful information, it is advised to keep preprocessing processes to a minimum. Preprocessing techniques such as part-of-speech tagging, lemmatization, stop-word removal, sentence segmentation, paragraph construction, tokenization, lowercase, and removal of punctuation are applied during the preprocessing step [19,79]. The objective of these procedures is to extract pertinent linguistic elements for plagiarism analysis and standardize the content [80–82]. To provide consistency in the text representation, for example, the lower casing is used to transform all characters to lowercase [83,84].

Because stop words and punctuation removal have such strong effects on word co-occurrences and, consequently, the corresponding relationships, they can significantly alter the resultant word embeddings [85]. For instance, in the phrase “*The regions of Earth* surrounding the Equator *are* known as the tropics.” taken from the English Wikipedia, look at the terms “tropics” and their contemporaries in a window size of five: The areas of Earth that surround the equator are known as the tropics. The italicized terms in this sentence were used about the word “tropics.” The sentences that follow demonstrate how eliminating punctuation and stopwords can alter the meaning of the term “tropics". The tropical areas of Earth that encircle the equator. The tropical areas of Earth encircle the equator. Although the removal of punctuation in this case has less of an impact than the removal of stopwords, we can still see that the word “Equator” is not the same as the other, indicating that punctuation can alter a word. A corpus contains a lot of punctuation, thus removing anyone can significantly alter the word’s context [84,86].

Identifying the limits of sentences or other language units inside the text is the main goal of segmentation [87–89]. To lessen the influence of particular numerical information on plagiarism analysis, numbers might be removed or replaced with placeholders. Furthermore, named entity recognition (NER) can provide important text by classifying and identifying named entities [90,91]. Eliminating common words with no semantic significance, such as “the,” “is,” and so on, is the process of removing stop words [92].

After the text has been purged of all punctuation and numerals, separated into the necessary tokens, and stop words eliminated, you can start transforming the words that remain. Eliminating affixes that is, components that are joined to the root and result in the creation of a new word is the next stage. Stemming and lemmatization [88] are two methods that can be used to accomplish this task; however, they differ greatly in terms of speed and transformation technique. Stemming is the process of removing prefixes, ends, and suffixes from a word so that the remaining portion remains the same in all incarnations of the word. Here, two issues arise under-stemming and over-stemming, which occurs when two terms with similar meanings are transcribed into two distinct forms, respectively, and two instances of different meanings are merged into one form [93]. The text dictionary can be made smaller by using one of the several stemming algorithms that have been developed to reduce words to their roots. Lovins stemming, Porter, Peisa-Huska, Dawson, HMM, and YASS are a few of these [94]. By giving each word a grammatical category (noun, verb, adjective, etc.), PoS tagging facilitates syntactic analysis and helps spot possible plagiarism by pointing out word use similarities [34,95,96]. Natural Language Processing (NLP) libraries provide reliable and practical tools to do these preparatory procedures [97–99]. In their multilingual and multi-functional text processing pipelines for plagiarism detection investigations, researchers mostly employ these libraries [100,101].

### Methods for extracting features

In natural language processing (NLP), the extraction of distinctive characteristics is crucial in facilitating efficient representation and analysis of textual input. Sentiment analysis, machine translation, text summarization, and other applications benefit from its ability to capture linguistic features, semantic information, and structural patterns [102]. Deeper linguistic analysis is made possible by extracting significant characteristics, which increases natural language processing application accuracy and productivity. The process of selecting and converting unprocessed data into a set of relevant features that accurately represent and explain the data is known as feature extraction [103]. Feature extraction is a subfield of natural language processing (NLP) that specializes in obtaining relevant and significant characteristics from textual input. In this procedure, words are taken out of the text data and transformed into features that classifiers may use [104]. Feature extraction reduces the volume of data by identifying the most valuable characteristics by merging variables into components. Plagiarism detection relies heavily on feature extraction. The text is transformed into numerical representations with relevant data using a variety of techniques and methods [105].

### Lexical

Finding linguistic distinctions between a language’s dialects frequently calls for in-depth human investigation and specialized knowledge. This is mostly because learning different dialects involves a great deal of intricacy and subtlety [106]. When calculating similarity, lexical identification techniques only take into account the characters present in a given text. The lexical detection techniques need to be used in conjunction with more advanced NLP techniques to identify obfuscated plagiarism [107,108]. Lexical detection techniques are also useful for detecting homoglyph substitutions, a prevalent way that technology disguises itself. Approaches to lexical detection often fall into the categories that are described in the next sections.

#### N. grams.

Character-level N-grams, which are N-character sequences, provide a deeper examination of the text’s structure [109,110]. On the other hand, word-level N-grams provide information about the syntactic and semantic patterns of the document by encapsulating N-word sequences. N-grams are typically represented numerically for quantitative calculations and comparisons. After representation, the N-grams are compared and contrasted to search for trends and variations [111,112]. The phrase “The cow jumps over the moon” is one example. If N=2 is referred to as bigrams, the N-grams would be:

- the cow;
- the cow leaps;
- the cow jumps over;
- over the moon.

Character n-gram comparisons can be utilized for cross-language plagiarism detection (CLPD) in cases when the languages involved such as Spanish and English have a high degree of lexical similarity [113].

#### Querying search engines.

Web search engines are used by many detection techniques for candidate retrieval, or the first step of the detection process, which is the identification of possible source documents. The success of this approach depends on the mechanism used to choose the query terms from the suspicious document [114] For example, finding the longest sentence in a paragraph and its keywords.

#### Vector space model (VSM).

The Vector Space Model has been widely used by researchers as a foundational method in plagiarism detection [10,11]. The VSM technique converts textual data into numerical vectors to represent texts and evaluate their similarity. This method makes it easier to do quantitative analysis, which makes it possible to find possible plagiarism cases. The fundamental concept of VSM states that every document is represented as a vector in a multi-dimensional space, with each dimension denoting a distinct word or term [115]. The significance or frequency of a term inside the document itself is indicated by the value of each dimension. N-gram words usually define the vector space’s dimensions in plagiarism detection, and each vector’s constituents are weighted according to the Term Frequency - Inverse Document Frequency (TF-IDF) relationship [116]. Inverse Document Frequency (IDF) values are derived from the dubious document or the corpus. The cosine measure is frequently utilized to measure the degree of similarity between vector representations; in other words, the angle that the vectors form acts as a proxy for the degree of similarity between the documents that the vectors represent. Here, lexical and grammatical traits are extracted and classified using tokens instead of strings. The similarity can be determined using a variety of vector similarity metrics, such as the Manhattan, Euclidean, Dice, Overlap, Cosine, and Jaccard coefficients [117]. It has been observed that the Cosine and Jaccard coefficients can be utilized to ascertain the degree of similarity between two vectors. Use the cosine coefficient to identify partial copying without revealing the content of the document. Thus, in situations where work submission is quite secret, this approach aids in the detection of plagiarism.

#### Stylometric features.

In the field of intrinsic plagiarism detection, researchers have frequently used stylometric-based feature extraction algorithms to examine the subtle differences in writing styles found in textual data [118]. To create a stylometric profile specific to every document, these characteristics are carefully measured [119]. Calculating statistical measurements such as word frequencies, average sentence lengths, or punctuation mark distributions is a common approach in stylometric feature extraction [120]. These metrics provide information on the writing style and preferences of the author.

### Semantic

Semantics-based approaches work under the premise that the presence of similar semantic units in two passages determines their semantic similarity [77]. Two units are semantically comparable when they occur in similar situations [120]. The fact that units with similar contexts tend to have higher semantic similarity is the source of the concept of semantic similarity. Many techniques make use of thesauri like WordNet or EuroVoc to take advantage of semantics in the study. The performance of paraphrase identification is improved by these thesauri’s useful semantic properties, which include synonyms, hyponyms (subordinate terms), and hypernyms (superordinate terms) [111].

#### Latent semantic analysis (LSA).

Using a matrix that has rows for words, columns for documents, and matrix components that typically reflect log-weighted Term Frequency - Inverse Document Frequency (TF-IDF) values, Latent Semantic Analysis (LSA) computes a matrix [121,122] to measure the similarity of term distributions in texts. The term-document matrix is then approximated at a lower rank by LSA using dimensionality reduction techniques like Singular Value Decomposition (SVD). To do this, fewer rows must be used while maintaining the distribution of column similarity. The phrases that survive the dimensionality reduction are thought to be the most representative of the semantic significance of the text. Consequently, by comparing the texts’ rank-reduced matrix representations, one may determine the texts’ semantic similarity [123]. Text similarity that conventional vector space models are unable to convey. Latent Semantic Analysis (LSA)’s capacity to handle synonymy is advantageous for paraphrase recognition.

#### Word embeddings.

One popular approach to intrinsic plagiarism detection is word embeddings-based feature extraction, which aims to extract intricate contextual and semantic meanings of words in a document. This method carefully places related words closer together by encoding words as dense vectors inside a continuous vector space [124]. It is possible to extract important features that reflect the semantic linkages found inside words thanks to this proximity-based representation. The first step in the procedure is to train a word embedding model on a large text corpus. By carefully examining the situations in which words are used, the model learns how to encode the semantic meaning of words throughout this training phase [125]. Word2Vec or GloVe, create these embeddings using methods like co-occurrence matrix factorization or skip-gram [112].

#### Graph-based semantic analysis.

A text is represented by a weighted directed graph in knowledge graph analysis (KGA), where the edges in the graph denote the relationships between the semantic concepts that the text’s words communicate, and the nodes represent the semantic concepts themselves [126]. Usually, the relations come from publicly accessible corpora like WordNet or BabelNet8. The main difficulty with KGA is figuring out the edge weights. In the past, WordNet’s idea relationships were examined to determine edge weights [127,128]. The weighting process was made better by Salvador et al. [129]by utilizing continuous skip-grams that also take the concepts’ context into account. Semantic similarity scores for documents or portions of phrases are obtained by applying graph similarity metrics.

### Syntax

By using PoS tagging to identify a sentence’s syntactic structure, syntax-based detection techniques normally function at the sentence level. By comparing only word pairs that correspond to the same PoS class, syntactic information might mitigate morphological ambiguity during the lemmatization or stemming step of preprocessing or reduce the workload of future semantic analysis [130]. PoS tag frequency is a stylometric parameter that is used in many detection strategies. Unlike lexical-based approaches, which focus on vocabulary and word usage, syntax-based approaches look at the syntactic structure and arrangement of sentences inside a document [131]. These characteristics can offer insightful information about the structure and content of the text, which can help spot possible plagiarism.

#### Syntactic.

One important aspect of intrinsic plagiarism detection is syntactic-based feature extraction, which is devoted to identifying the grammatical structure and structural blueprints of text [121]. This method delves into the complex syntax that determines sentence structure inside a document, going beyond simple lexical considerations. In addition, a variety of syntactic elements are utilized, including phrase structures and dependency interactions. For example, dependency relations record the syntactic interactions among words; they include subject-verb-object relationships and provide information on sentence structure and similarity [80]. Analyzing phrase structures both verb and noun phrases helps one comprehend how syntactic alignment works within a sentence. These characteristics work together to make it easier to compare and recognize similar or paraphrased phrases, which strengthens the detection of plagiarism that is based on syntactic tricks [132].

#### POS tagging.

One of the sequence labeling tasks is part-of-speech (POS) tagging, which entails giving each word a grammatical category label based on contextual and linguistic information [133]. A word’s tag or label offers details about the word and the lexical categories that surround it. A POS tagger would typically divide a sentence into several subcategories according to the parts of speech it contains, such as nouns, pronouns, adjectives, verbs, adverbs, and so on. Because they offer linguistic information on how words can be used in a phrase, sentence, or document, POS tags are useful. For many Natural Language Processing (NLP) frameworks, POS tagging is an essential preprocessing step in the language processing industry, Speech recognition, sentiment analysis, question answering, chunking, Named Entity Recognition (NER), word sense disambiguation, and more are examples of these NLP frameworks [134]. It can be difficult to determine a language’s grammatical class since it changes according to the context in which it is employed.

As a result, it can be challenging to tag every word in a phrase when some words have many grammatical POS labels. The issue of POS tagging has been extensively studied in English, several European languages, and most South Asian languages [34]. Research on Indian languages is still necessary, though, especially on the Odia language, since it can be difficult to study languages with complex morphological inflection and variable word order. Additionally, POS tagging in Odia is made more difficult by the absence of capitalization, gender information, and other elements. To determine each input word’s distinct grammatical POS, a POS tagging technique is required. The POS tagging task is approached using a variety of algorithms, including rule-based, probabilistic, deep learning, and hybrid techniques [135,136].

### Plagiarism detection techniques

#### Traditional techniques.

Apart from the widely employed methods in intrinsic plagiarism detection, several conventional approaches have demonstrated potential in this domain.

1. Style Change Function: When detecting suspected plagiarism in a text that has unreported changes in writing style, stylistic analysis is a crucial component of the process [69]. In this paper [54] by Hourrane O, et al. (2019), the intrinsic plagiarism detection problem is approached using several embedding types. Architecture is used in two sub-tasks in this study. The first is style change detection, in which the writers check to see if an input document has sections produced by different authors because of style changes. The second method involves the writers identifying any obtrusive passages that stylistically depart from the primary writing style. This is known as style breach detection.
2. Lucene for Indexing: One popular indexing library that may be used to effectively store and retrieve textual data is Lucene, which can also be used to compare and identify passages that have been copied [137,138].

#### Statistical techniques.

When detecting intrinsic plagiarism, statistical and distance-based methods are frequently employed to gauge how similar or distinct two text texts are. To measure how much linguistic traits, word frequencies, or stylistic patterns coincide or diverge, these methods use a variety of statistical metrics, including hashing, character and n-gram profiles, and frequency distance.

1. Frequency Difference: It is now possible to compare text segments and identify writing style differences thanks to the usage of distance metrics like frequency difference and pq-gram distance [139]. Furthermore, these methods have demonstrated potential in the area of cross-language plagiarism detection, as they have proven successful in determining the degree of textual similarity between languages. A method [140] for identifying text alignment between the suspicious and source documents was presented by El-Rashidy et al. in 2022. Their main contribution is the term frequency-inverse sentence frequency (tf-isf) method, which is used to identify instances of plagiarism in sentences.
2. Lempel-Ziv Compression: Style change functions and Lempel-Ziv compression with their various approaches to identifying and evaluating stylistic variances in text documents, these techniques each contribute something new to the discipline. Furthermore, new methods of identifying segments with distinct writing styles and detecting plagiarism have been made possible by the addition of compression and style change tools [141].

#### Distance based techniques.

To identify possible cases of plagiarism, statistical and distance-based methodologies quantify stylistic differences and similarities within a manuscript.

1. Principal Component Analysis (PCA) with Distance Score: An innovative method for intrinsic plagiarism detection is presented in [142] by Veisi et al. in 2022. It suggests creating vectors of character trigram frequencies to represent the successive windows that make up a suspicious document. After that, an altered normalized distance measure is used to create the distance matrix to compare each window with the others.
2. Character N-Grams Profile Method: Bensalem et al. considerably advance the field in their study [143,144] by enhancing the method’s parameters and incorporating larger feature sets. The technique makes use of a dissimilarity metric that was initially created for author identification as well as character n-gram profiles. Furthermore, heuristic principles are put forth to help identify passages that have been copied, find texts that are free of plagiarism, and lessen the effect of unrelated style modifications.

#### Methods of supervised machine learning.

These methods include the use of word embeddings in embedding-based approaches, stylistic feature-focused stylometric analysis, and linguistic analysis with n-gram frequencies [145]. Machine learning algorithms facilitate the identification of potentially plagiarised passages within a manuscript as well as changes in writing style. Furthermore, by offering important insights into identifying and avoiding plagiarism in textual content, these ML algorithms significantly improve the precision and efficacy of intrinsic plagiarism detection systems [138].

1. Support Vector Machine: Three stages comprise the implementation of the proposed system [19] by the authors El-Rashidy et al., (2024). Preprocessing techniques include part-of-speech labelling, lemmatization, lower casing, stop-word removal, punctuation removal, eliminating all tokens that don’t begin with a letter, sentence segmentation, paragraph composition, and tokenization. Second, the training database is created, the support vector machine model is constructed, lexical, syntactic, and semantic features are computed, the collection of potentially plagiarised cases is extracted, and the most valuable features are selected using the seeding technique [71]. The sentence similarity cases are found using the first path, which is based on a traditional paragraph-level comparison, and the second path, which is based on the hyperplane equation of the constructed SVM classifier. In the last stage, approaches like as adaptive behavior, filter segments, filter seeds, and merging adjacent identified seeds are used to extract the best-plagiarized segment between suspicious and source texts.
2. Decision Tree: In supervised learning, Decision Trees (DTs) are predictive models that are well-known for their resilience, interpretability, and undeniable usefulness across a broad spectrum of applications. To address the three fundamental objectives of a predictive learner—fitting training data, generalization, and interoperability. This study [146] by the authors, Costa and Pedreira, (2023), offers an overview of the most important recent developments in DT research. Decision Trees (DTs) are widely used learning models. Typically, these models are shown as a structure akin to a flowchart, where each internal node represents a split or logical test, and each leaf represents a prediction.
3. K Nearest Neighbor (KNN): On the smaller dataset, KNN, SVM, and DT were the classification techniques employed by Eppa and Murali (2022) in the study [147]. The next sections go into detail about how these algorithms are implemented as well as the accuracy that can be achieved with them. The seven closest neighbors and the weights allocated depending on distance were taken into account when implementing the K Nearest Neighbours algorithm [148]. The algorithm used for the Decision Trees Classifier was based on the Gini Index.
4. Deep Learning: Plagiarism detection can be accomplished using a variety of deep learning methods. A few of them are enumerated below:
  (a) Convolutional Neural Network (CNN): By utilizing CNNs with parameters Adaptive Moment Estimation (ADAM), Stochastic Gradient Descent (SGD), and Root Mean Square Propagation (RMSProp), we present a unique approach for authorship verification in Urdu that closes this gap. To support the development of this approach, we have put together a new corpus, called UAVC-22, that is intended for Urdu authorship verification. This corpus offers enhanced robustness concerning author and separate word classes. Word2Vec, GloVe, and FastText are three different text embedding techniques that are used. Nine authorship verification models have been developed by this study [149]. We have compared the CNN-based method [150,151] to more traditional machine learning models like SVM and RF to assess its efficacy and superiority. For the Urdu dataset UAVC-22, the CNN-ADAM model that was optimized with FastText got the greatest accuracy of 98%.
  (b) Long Short-term Memory (LSTM): The limited resources available to low-resource text plagiarism detection provide a considerable barrier to labeled data that is available for training. The creation of complex algorithms that can recognize patterns and distinctions in texts is necessary for this endeavor, especially in the fields of translation-based plagiarism detection and semantic rewriting. We present an enhanced attentive Siamese Long Short-Term Memory (LSTM) network [115] in this work [152], which is designed to detect Tibetan-Chinese plagiarism. As part of our plan, we first introduce translation-based data augmentation to expand the bilingual practice data set. Subsequently, we offer a pre-detection approach that leverages abstract document vectors to enhance detection efficiency. Lastly, we present an enhanced Siamese LSTM network designed for detecting plagiarism in Tibetan and Chinese. We carry out extensive tests to demonstrate the efficacy of our suggested methodology for detecting plagiarism.
  (c) Bidirectional Long Short-Term Memory Network (BiLSTM): The recommended algorithmic method for extracting information locates text lines within a given document by utilizing deep learning classification techniques that characterize the metadata of the proposed algorithmic PC. We particularly used two BiDirectional LSTM architectures [153] and a character convolutional neural network to categorize each sentence in an article as either an algorithmic metadata group or a class unrelated to algorithms. Even if word embedding-based techniques are appropriate for domain-specific tasks, structural parsers still need to manage a variety of issues, including morphological shifts and indeterminate segmentation, which makes obtaining prior knowledge costly. Text comprehension is largely tailored to a single language; numerous rules need to be created from scratch if a language changes.

#### Methods for unsupervised machine learning.

These methods seek to locate groups of writers that share a common writing style and find any discrepancies that might point to possible plagiarism.

1. K-Means: Only pertinent documents could be selected for detection by clustering the documents using an unsupervised machine learning method such as K-means [154]. The TFIDF text encoding strategy calculates the degree of similarity between the suspicious article and the corpus of source articles using NLP, K-means clustering, and cosine similarity approaches [155].
2. Agglomerative Hierarchical: The development of AI technology has made content creation simpler and more widely available. It is challenging to distinguish between text produced by AI and text produced by humans in such a situation. Our approach suggests an intelligent system that may use stylometric analysis to identify distinctive writing styles from text files to allay this worry. Using silhouette scores as performance indicators, this paper also examines several clustering methods [156], such as k-means, k-means++, hierarchical, and Density-Based Spatial Clustering of Applications with Noise (DBSCAN). This guarantees that our system will be successful in differentiating between similar writing styles and those that are not based on sophisticated linguistic and structural aspects of the text. Our technologies gather text of different styles together and separate it, offering a useful way to detect plagiarism across several document files [157].
3. Density-Based Spatial Clustering of Applications with Noise (DBSCAN): Based on how it is detected, plagiarism can be divided into two main categories: intrinsic and extrinsic plagiarism. In contrast to intrinsic plagiarism detection, which uses writing style variance to identify plagiarism without the use of a reference corpus, extrinsic plagiarism detection compares a text to a predetermined reference dataset. While there are numerous methods for identifying extrinsic plagiarism, there aren’t many for identifying intrinsic plagiarism. This work [158] by Saini et al., 2021 presents a streamlined methodology for creating a plagiarism detector that can effectively identify instances of plagiarism even in the absence of a reference corpus. The method focuses on creating a plagiarism detection system by employing DBSCAN clustering [159] and stylometric features to determine the authors’ writing styles inside the article. The user-friendly interactive interface of the proposed system allows the user to upload a text document to be examined for plagiarism, and the results are shown directly on the web page. Furthermore, the user has access to graphs that represent the document’s analysis.

## Evaluation techniques

Researchers in NLP and Information Retrieval (IR) need access to datasets for development and assessment. The PAN datasets are a thorough and reputable forum for comparing plagiarism detection systems and approaches [67]. The PAN test datasets include synthetic examples of cross-language plagiarism and artificially generated monolingual instances with varying degrees of obfuscation. The majority of the studies in this survey that describe algorithms for lexical, syntactic, and semantic detection make use of the Microsoft Research Paraphrase corpus or PAN datasets. Since detecting plagiarism involves retrieving information, approaches for evaluating plagiarism detection are commonly based on precision, recall, and F-measure [67]. While language dependence affects the diversity of detection targets, abstraction level affects both detection accuracy and generality. Efficiency determines how quickly and efficiently resources are detected, while extensibility guarantees application across a range of project sizes and contexts. These elements are essential to software development and maintenance because they determine the quality and usefulness of code similarity detection [160].

A variety of criteria that offer information about these algorithms’ performance are used to evaluate intrinsic plagiarism detection techniques[161]. These metrics include clustering evaluation criteria, classification-based measurements, and metrics created especially for activities involving the detection of plagiarism [19]. The following classification measures are frequently used: F1 Score, Accuracy, Precision, and Recall. Precision calculates the percentage of genuine positives among expected positives, whereas Accuracy measures the total correctness of forecasts. For datasets that are not balanced, the F1 Score provides a balance between recall and precision [80]. A statistic called granularity is used in clustering techniques to assess how detailed the clustering results are. It demonstrates the clusters’ fine graininess. Furthermore, an assessment criterion called Plagiarism Detection, or PlagaDet for short, assesses how well clustering techniques perform when it comes to plagiarism detection. Furthermore, metrics designed specifically for comparing clustering partitions include WindowDiff, WindowP, WindowR, and WindowF. Finally, a statistical measure called mean distance calculates the typical separation between data points inside clusters [28,69].

## Findings and trends

Due to the abundance of material available on the internet and the strength of search engines, plagiarism is becoming a severe issue in many fields, including education. Usually, plagiarism is separated into two types: intentional and inadvertent. However, techniques for detecting plagiarism can also be applied in other domains, such as information retrieval, where a text is input and the most appropriate matches are identified. Systems for detecting plagiarism are useful not just in the sectors of education and information retrieval, but also in publishing, research, and litigation. In publication as well as research, it is essential to guarantee the authenticity and uniqueness of published work. Plagiarism detection technology can prevent academic misconduct and maintain the standard of published writing by identifying instances of repetition or similarity with previously published works. In legal proceedings, plagiarism detection can be used to identify instances of intellectual property theft or copyright infringement.

Over time, plagiarism detection algorithms have undergone significant evolution, utilizing various approaches to address the difficulties of detecting stylistic modifications and possible instances of plagiarism in a given work. Early research focused on distinguishing original from plagiarised content by quantifying stylistic features [118] and employing traditional discriminant analysis [122,138]. The character n-gram profiles approach was developed [109,110], using character-based n-gram features for detection. Principal component analysis was employed in the same year by PCA with distance scores [142] to identify stylistic variations according to distance metrics. The use of Lempel-Ziv compression [80,141], which makes use of compression algorithms to identify inconsistencies and stylistic shifts, represents a further advancement. The same year, the idea of style change functions [54,69] was put forth to detect changes in a document’s writing style. To organize related text segments according to stylistic similarities, clustering algorithms including Agglomerative Hierarchical clustering [156,157] and K-Means clustering [154,155] were used. Subsequent methods like Transformer models [18], Decision Trees [146], and Support Vector Machines [19,71] contributed significantly to the field. Random Forest, AdaBoost, Multilayer Perception (MLP), and LightGBM ensemble learning [162] demonstrated the advantages of mixing several models for better results. LSTM + BERT [21], and GAN-based encoder and decoder [163] are examples of recent developments.

**Table 3:** List of abbreviations.

Term	Full Name	Term	Full Name
AI	Artificial Intelligence	HC3	Human ChatGPT Comparison Corpus
NLP	Natural Language Processing	LLMs	Large Language Models
PD	Plagiarism Detection	PAN	Plausible Argumentation and Negotiation
GPT	Generative Pre-trained Transformer	SJR	Scientific Journal Ranking
SVM	Support Vector Machine	CEN	Corpus of English Novels
BERT	Bidirectional Encoder Representations from Transformers	XML	Extensible Markup Language
CTRL	Conditional Transformer Language Model	NER	Named Entity Recognition
XLM	Cross-lingual Language Model	PoS	Part of Speech
XLNET	Generalized Autoregressive Pretraining for Language Understanding	VSM	Vector Space Model
PPLM	Plug and Play Language Model	tf-idf	Term Frequency- Inverse Document Frequency
HWT	Human-Written Text	IDF	Inverse Document Frequency
RNN	Recurrent Neural Network	LSA	Latent Semantic Analysis
LSTM	Long Short-term Memory	SVD	Singular Value Decomposition
PCA	Principal Component Analysis	KNN	K Nearest Neighbor
CNN	Convolutional Neural Network	ADAM	Adaptive Moment Estimation
RMSProp	Root MeanSquare Propagation	SGD	Stochastic Gradient Descent
BiLSTM	Bidirectional Long Short-Term Memory Network	DBSCAN	Density-Based Spatial Clustering of Applications with Noise
MLP	Multilayer Perception	IR	Information Retrieval

In recent times, there has been a noticeable increase in the performance of large language models (LLMs) in several tasks [164]. The increasing prevalence of plagiarism in academic writing is a significant concern linked to the increasing reliance on ChatGPT [11]. This might potentially jeopardize the objective and integrity of assignments and tests. Various AI-generated text classifiers and tools, like Log Likelihood [165], RoBERTa-QA (HC3) [44], GPTZero [11], OpenAI Classifier [14], DetectGPT [58], and Turntin [166] Plagiarisma, Plagiarismdetect, Duplichecker, Grammarly, PlagAware, Quetext, PlagScan [28], have been developed by researchers to lessen the potential for plagiarism resulting from the use of LLMs. To enhance comprehension, a list of abbreviations is provided in Table 3.

## Challenges in plagiarism detection

We have examined several papers on plagiarism identification [19,69,139,142]. There is a dearth of research on the detection of plagiarism in tables and figures in natural language, and the technologies now in use are unable to identify plagiarised images, tables, figures, formulas, and scanned papers. The security and privacy of the technologies provide additional difficulty. Certain tools save the documents that users provide in their repository. One well-known commercial tool that stores student papers and assignments in its database for potential plagiarism detection in the future is Turnitin. It is regarded as an unlawful activity [28].

1. The lack of a reference list or reliable sources for comparison is one major obstacle. It is challenging to differentiate between cases of plagiarism and true stylistic modifications due to the absence of external references [116].
2. The diversity and intricacy of writing styles present another difficulty. Different writing styles can be used by authors either purposefully or accidentally, which can result in variances in their work [119].
3. The other difficulty in intrinsic plagiarism detection is identifying the best attributes and representing them. The writing style has been studied using a variety of criteria, including word frequencies, grammatical structures, character n-grams, and semantic patterns [28].
4. Plagiarised passages that are scattered or broken up across a manuscript can be considered instances of intrinsic plagiarism. These partial plagiarism cases, in which just particular sentences or phrases are replicated, call for sophisticated algorithms capable of detecting minute variations and parallels in the textual content of the document [150].
5. Because plagiarism writers might use complex techniques to hide or modify their writing styles, it becomes more challenging to identify cases of plagiarism based only on inherent traits [80].
6. Another issue is the absence of linguistic heterogeneity and diversity among languages with limited resources. The variety of dialects, registers, and writing styles seen in high-resource languages add to the language’s complexity and diversity [90].
7. Plagiarism detection in low-resource languages is further complicated by the lack of language-specific stylometric cues. Writing style is mostly captured and quantified by stylistic elements including word frequency, n-grams, and grammatical patterns.
8. The scarcity of corpora and reference materials for languages with little resources is another issue. For referencing and contrasting, scholars working in high-resource languages, have access to extensive databases of texts, books, and internet resources. These resources, however, could be hard to come by, insufficient, or difficult to access digitally in languages with limited resources [167].
9. Report generation and processing take a lot of time, which presents difficult issues. The larger the document, the longer it takes and the more bandwidth it requires. Because of its extensive feature set and large user base, Turnitin is regarded as one of the best plagiarism detection tools [28].
10. Significant obstacles in the detection of plagiarism also stem from technical limitations and restrictions. Although they don’t need to be downloaded and installed on a user’s computer, web-based tools still need a fast internet connection. A finite number of documents with a finite file size can be handled at once using the current tools. They take a long time to review a large number of documents [28].

## Conclusion

Detecting plagiarism is a crucial area of text analysis that searches a document for instances of duplicate content and establishes whether or not the same author wrote a piece of the text. With the advent of large language model-based content creation systems like ChatGPT that are accessible to the public, the issue of intrinsic plagiarism has become more significant in a variety of businesses. Because computers are available and used in classrooms, and because electronic information on the internet is typically widely accessible, more and more students are plagiarizing. To cope with this shifting environment, there is a growing need for accurate and dependable detection techniques. This study examines the efficacy of various plagiarism detection methods and compares their ability to discern between information produced by artificial intelligence (AI) and human-generated content. To give readers a general picture of the state of the research on computational techniques for plagiarism detection, this article thoroughly assesses 189 research papers that were published between 2019 and 2024. We suggest a new technically focused framework for attempts to prevent and identify plagiarism, academic plagiarism types, and computational techniques for detecting plagiarism to organize the way the research contributions are presented. We show that there is a wealth of active research on the subject of plagiarism detection. During the period we reviewed, a great deal of progress has been achieved in the field of automatically recognizing plagiarism that is highly hidden and therefore challenging to detect. The exploration of features of non-textual content, the use of machine learning, and improved techniques for semantic text analysis are the key forces behind these breakthroughs. Our analysis leads us to the conclusion that the combination of several analytical methodologies for textual and characteristics of non-textual content is the most potential area for future research contributions to further enhance plagiarism detection.

## Future research directions

As technology advances, we recommend that authors use graph-based structures, images, references, and citations in their future work to further boost the detection process. We want to use deep learning and graph clustering approaches in conjunction with graph-based structures to identify plagiarism in the future.
